# Enhanced Electrical Performance and Stretchability by Plasticizer‐Facilitated PEDOT:PSS Self‐Alignment

**DOI:** 10.1002/advs.202502853

**Published:** 2025-05-08

**Authors:** Carla Volkert, Mateusz Brzezinski, Pablo Gomez Argudo, Renan Colucci, Sapun H. Parekh, Pol Besenius, Jasper J. Michels, Ulrike Kraft

**Affiliations:** ^1^ Organic Bioelectronics Research Group Max Planck Institute for Polymer Research 55128 Mainz Germany; ^2^ Department of Molecular Electronics Max Planck Institute for Polymer Research 55128 Mainz Germany; ^3^ Department of Molecular Spectroscopy Max Planck Institute for Polymer Research 55128 Mainz Germany; ^4^ Department of Biomedical Engineering University of Texas Austin Austin TX 78712 USA; ^5^ Department of Chemistry Johannes Gutenberg University Mainz 55128 Mainz Germany

**Keywords:** chain‐alignment, PEDOT:PSS, plasticizer, stretchable electronics, Flory‐Huggins

## Abstract

Stretchable, soft electronics have high potential for wearable healthcare applications and biointerfacing. One approach to render inherently brittle conductive polymers such as poly(3,4‐ethylenedioxythiophene):poly(styrenesulfonate) (PEDOT:PSS) stretchable are organic plasticizers. However, little is known on how they affect the morphology and in result the electrical properties of conductive thin‐films. This study fundamentally explores this relationship using a bilayer model of transfer‐printed PEDOT:PSS on stretchable, biocompatible poly(vinyl alcohol) substrates infused with glycerol (15–55 wt.%). The diffusion of the plasticizer leads to a reorganization of PEDOT and PSS, which is investigated using a multicomponent diffusion model. This approach correctly predicts the (plasticizer‐dependent) increase in conductivity that followed plasticizer diffusion and is attributed to the reorganization toward more interconnected PEDOT domains. In result, the system shows an improved electrical response to strain as well as crack‐free elongation. Simultaneously, the electrical resistance decreases to one‐fifth of its initial value, which is attributed to chain‐alignment upon strain.

## Graphical abstract

To advance stretchable electronics, understanding how organic plasticizers influence poly(3,4‐ethylenedioxythiophene):poly(styrenesulfonate) (PEDOT:PSS) morphology and conductivity remains crucial. A bilayer model with glycerol‐infused poly(vinyl alcohol) (PVA) substrates reveals that plasticizer diffusion reorganizes PEDOT and PSS, improving conductivity and strain response. This results in crack‐free elongation and a fivefold reduction in resistance due to chain alignment.

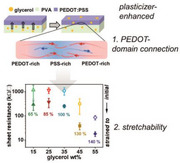

## 1 Introduction

Significant potential lies in flexible and stretchable electronics with applications ranging from portable electronics over wearable devices on‐skin electronics and neuroprosthetics to personalized healthcare. As a result, extensive research efforts are directed toward the development of conformable devices, including biosensors,^[^ ^1^ ^]^ optoelectronic devices,^[^ ^2^ ^]^ organic field effect transistors (OFETs),^[^ ^3^, ^4^ ^]^ light‐emitting diodes (LEDs)^[^ ^5^ ^]^ and organic electrochemical transistors (OECTs).^[^ ^6^, ^7^, ^8^, ^9^, ^10^ ^]^ As flexible and stretchable electronics advance, particularly in healthcare applications, the quest for materials capable of maintaining their electrical properties under deformation becomes increasingly crucial. Various materials, including poly(3,4‐ethylenedioxythiophene):poly(styrenesulfonate) (PEDOT:PSS), have shown promise in creating devices for bioelectronics and personalized healthcare.^[^ ^11^, ^12^ ^]^ Beyond its biocompatibility, PEDOT:PSS offers high and tunable conductivity, transparency, and commercial availability.^[^ ^13^, ^14^ ^]^ However, the inherent brittleness^[^ ^6^, ^15^ ^]^ and limited elongation capabilities of pristine PEDOT:PSS (2%^[^ ^16^ ^]^) pose challenges for meeting the requirement of tolerating up to 30% strain for applications in healthcare electronics.^[^ ^8^ ^]^ To transform PEDOT:PSS from a brittle into a stretchable organic semiconductor, various PEDOT:PSS‐plasticizing approaches have been explored. These include the incorporation of plasticizing chemicals such as ionic liquids,^[^ ^17^, ^18^, ^19^, ^20^, ^21^, ^22^ ^]^ Triton‐X 100^[^ ^15^, ^23^, ^24^, ^25^ ^]^ and/or low molecular weight organic compounds.^[^ ^15^, ^26^, ^27^ ^]^ Additionally, blends of PEDOT:PSS with non‐conductive polymers as well as the addition of one or more additives to these blends have been investigated in previous reports.^[^ ^15^, ^18^, ^28^, ^29^, ^30^ ^]^ Efforts to stretch pristine PEDOT:PSS have been reported as well.^[^ ^31^, ^32^, ^33^ ^]^ The presented strategies enable PEDOT:PSS to maintain its conductivity to varying degrees during elongation and pave the way toward stretchable electronics. However, the underlying mechanism for PEDOT:PSS plasticization remains poorly understood. In addition to modifying the PEDOT:PSS formulation to enhance stretchability, diverse supporting substrates have been explored. These include polyimide (PI),^[^ ^32^, ^33^ ^]^ poly(dimethylsiloxane) (PDMS)^[^ ^19^, ^20^, ^33^, ^34^ ^]^ and styrene ethylene butylstyrene (SEBS).^[^ ^17^, ^21^, ^26^, ^31^, ^35^ ^]^ In the pursuit of stretchable healthcare electronics, we recently reported the usage of poly(vinyl alcohol) (PVA) as an attractive polymer for supporting substrates.^[^ ^36^ ^]^ It offers biocompatibility, mechanical softness, and high water content, making it suitable for medical applications.^[^ ^37^ ^]^

The striking need for novel approaches toward deformable conducting materials and supporting substrates is underlined by crucial material limitations such as insufficient stretchability and toxicity, which makes current systems unsuitable for medical applications. Furthermore, ensuring stable electronic properties is essential for bridging current technological gaps in deformable electronics. In addition, although plasticizers have been shown to allow PEDOT:PSS to better adapt to strain, little is known about the underlying mechanisms. In this work, using our previously presented transfer‐printing method,^[^ ^36^ ^]^ we fabricate a biocompatible bilayer system with pristine transfer‐printed PEDOT:PSS films on PVA:glycerol substrates. Within this system, glycerol is found to be capable of permeating into the conductive layer, triggering morphological rearrangement and subsequently inducing stretchability. Through a comprehensive analysis including multicomponent diffusion modeling on the plasticizer‐induced PEDOT:PSS rearrangement, electrical characterization, microscopic imaging, Raman spectroscopy and scanning force microscopy (SFM), we find that the diffusion‐active glycerol in the PVA substrate facilitates crack‐free elongation of PEDOT:PSS films (up to 160%). Furthermore, through chain‐alignment in PEDOT:PSS, its ability to maintain its electronic properties upon strain is significantly increased.

## 2 Results and Discussion

As described above, the underlying mechanism and plasticizer‐triggered rearrangements of PEDOT and PSS remain poorly understood. This is partly because these rearrangements usually take place in the liquid state during processing, but also because plasticized systems are inherently more mobile and therefore more difficult to study. Hence, we employed a model system consisting of PEDOT:PSS films on PVA substrates loaded with 15–55 wt.% glycerol to study the effect of an organic plasticizer on PEDOT:PSS in detail. We select this range as it balances capturing loading‐dependent effects on PEDOT:PSS while avoiding potential system interference from excessively high plasticizer concentrations.

**Figure 1:**
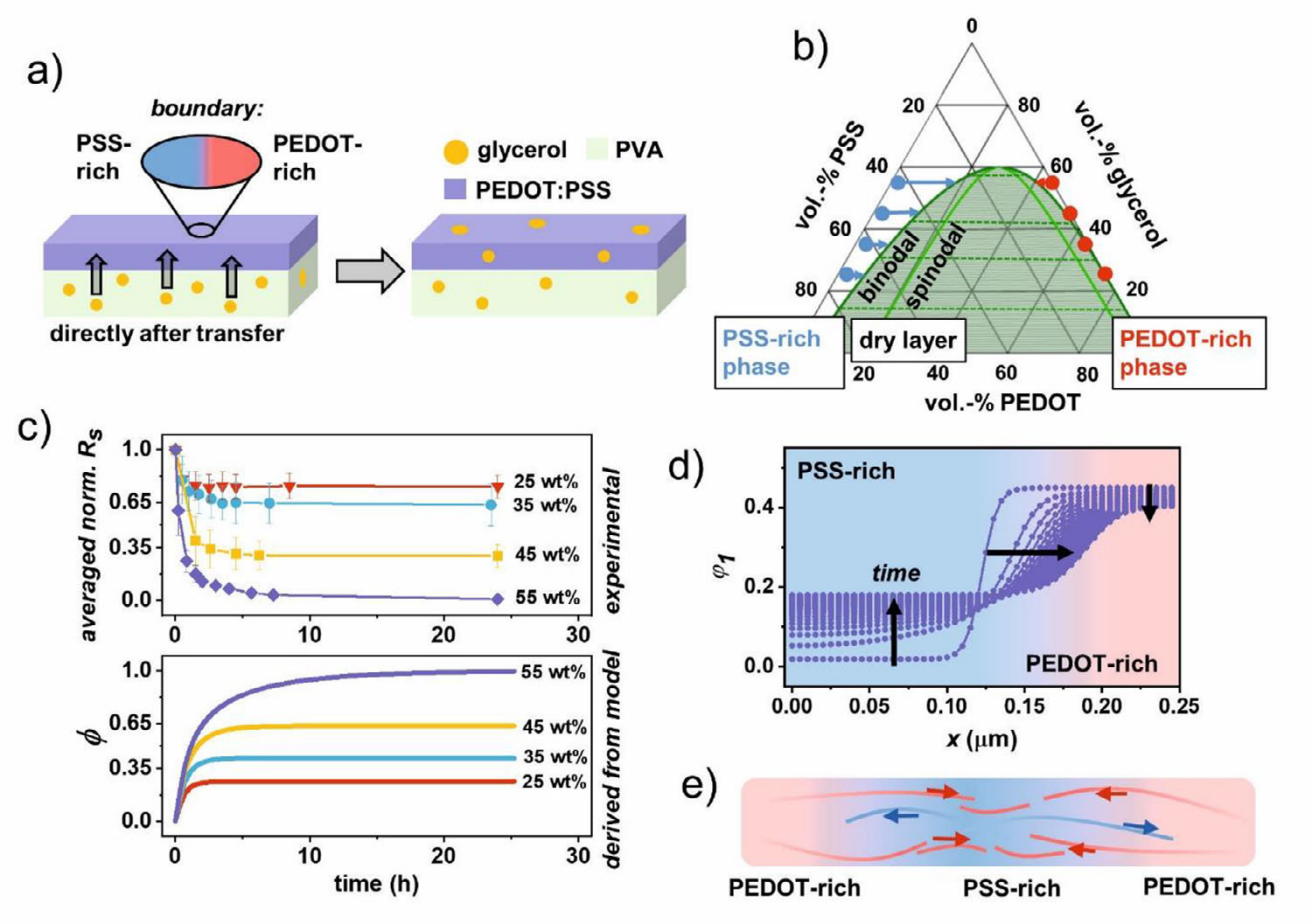
Diffusion‐active substrates: a) Schematic depicting glycerol diffusion from the substrate into the conducting PEDOT:PSS thin film. Magnified: Simplified boundary between PEDOT‐ and PSS‐rich areas in core‐shell structure. b) Calculated ternary phase diagram for the mixture PEDOT:PSS:glycerol. The green dashed lines are tie‐lines that connect compositions of coexisting phases. The blue and red dots indicate the initial out‐of‐equilibrium compositions after glycerol ingress. The arrows show the change in composition during polymer redistribution. The dashed green lines are tie‐lines connecting the compositions of coexisting phases. c) Top: Experimentally obtained decrease of the averaged normalized sheet resistances over time of transfer‐printed PEDOT:PSS on glycerol‐loaded PVA substrates. Bottom: Normalized fraction of PEDOT in the PSS rich phase (Φ). d) Concentration profiles of normalized PEDOT concentration versus spatial coordinate in a 250 nm 1D domain at different points in time for substrates containing 55 wt.% glycerol. e) Schematic drawing of the intermixing of PEDOT and PSS triggered by glycerol: More PEDOT migrates into the PSS‐rich phase than vice versa, which leads to better connected PEDOT grains.

The PEDOT:PSS films were uniformly spin‐coated on glass slides and subsequently transfer‐printed onto the PVA:glycerol substrates using our previously reported methodology.^[^ ^36^ ^]^ Since the glycerol is not covalently bound by either the PVA or the PEDOT:PSS, we hypothesized that after transfer‐printing of PEDOT:PSS films onto PVA:glycerol substrates, glycerol would partially diffuse from the substrate into the conducting thin film and partition across these two layers. This diffusion should be driven by a gradient in the glycerol's chemical potential between the substrate and the PEDOT:PSS thin film (schematically depicted in **Figure** **1**a). Due to the ingress of glycerol in the PEDOT:PSS film, we expected an increase in the conductivity^[^ ^26^, ^38^, ^39^ ^]^ (or decrease in resistance, respectively). Assuming that glycerol acts as a plasticizer we also anticipated an increased PEDOT:PSS polymer‐chain mobility, which would allow enhanced mechanical adaptation during stretching. We tested these hypotheses on increasingly glycerol‐loaded PVA substrates with data recorded for substrates containing 15 wt.% glycerol displayed in green, 25 wt.% in red, 35 wt.% in blue, 45 wt.% in yellow and 55 wt.% in purple throughout this study. Since the vapor pressure of glycerol is very low at room temperature,^[^ ^40^ ^]^ we can safely neglect any evaporation‐related changes in the glycerol content of the substrates.

First, the sheet resistance of the static PEDOT:PSS films is recorded as a function of time over a period of 24 h after transfer‐printing (Figure 1c upper panel) to equilibrate the system and to study the response of the PEDOT:PSS films to the partitioning of the glycerol. The Methods and Materials Section and Section S1 (Supporting Information) contain a detailed description of the sample preparation, as well as the absolute resistance plotted against the time for each set of films (Figure S1, Supporting Information). Except for the 15 wt.% glycerol films, the sheet resistance indeed slowly decreases over time, leveling off at lower values (higher conductivity) with higher glycerol percentages. This observation confirms the hypothesis that non‐conductive molecular plasticizers can diffuse from an underlying substrate into the conductive polymer layer thereby affecting its conductivity. Fitting the sheet resistance versus time data to an empirical exponential function (see Figure S2, Supporting Information) yields an apparent diffusivity of the order 10^−19^–10^−18^ m^2^ s^−1^. Since this very low value is not consistent with the diffusion of a molecule as small as glycerol (see Section S1.1., Supporting Information), the rate in the decrease in sheet resistance is much more likely determined by a redistribution of the bulky polymer chains within the PEDOT:PSS film, which occurs on significantly longer timescales, however, facilitated by fast initial ingress of glycerol. Furthermore, the dependence of the plateau value on the glycerol loading (Figure 1c) suggests that the final sheet resistance represents a thermodynamic equilibrium.

To better understand the reorganization of PEDOT and PSS triggered by the plasticizer glycerol, as well as and its dependence on the glycerol loading, a generalized 1D multicomponent diffusion model based on mixing thermodynamics was employed, while making the following assumptions: i) the PEDOT:PSS morphology consists of nano‐scopic well‐conducting PEDOT‐rich and poorly conducting PSS‐rich domains,^[^ ^32^, ^33^, ^41^ ^]^ of which the latter are the limiting factor for the overall conductivity of the blend, ii) PEDOT and PSS are mutually immiscible but glycerol acts as a compatibilizer that enhances intermixing and iii) the average concentration of glycerol in the PEDOT:PSS film increases with the loading of the PVA‐substrate, assuming a 1:1 partitioning. This last assumption is based on the fact that glycerol is highly polar and capable of both accepting and donating hydrogen bonds, which will form with either of the three media, be it PVA, PEDOT or PSS. It is hence reasonable to assume a high and similar glycerol uptake capacity for these substances, which is consistent with glycerol doping levels in PEDOT:PSS reported in the literature.^[^ ^38^, ^39^ ^]^ Furthermore, due to the fact that the PEDOT:PSS film is ∼1000 times thinner than the substrate, the change in glycerol concentration in the latter due to diffusion into the former is negligible. In our calculations, we consider the redistribution of PEDOT, PSS, and glycerol inside a nanoscopic “unit cell” containing a single interface (schematically depicted in Figure 1a). As we will show, with this coarse‐grained representation we can explain both the time‐dependence and the saturation of the normalized sheet resistance as a function of glycerol content without having to make speculations concerning the details of the PEDOT:PSS morphology. Within our model, the rate of change of the local volume fractions of PEDOT ($\phi_{1}$), PSS ($\phi_{2}$) and glycerol ($\phi_{3}$) is given by:

(1) $$\frac{\partial \phi_{1}\left(x\right)}{\partial t}=\frac{\partial }{\partial x}\;\Lambda_{11}\frac{\partial }{\partial x}\left(\frac{\delta F}{\delta \phi_{1}}\right)+\frac{\partial }{\partial x}\Lambda_{12}\frac{\partial }{\partial x}\left(\frac{\delta F}{\delta \phi_{2}}\right)$$

(2) $$\frac{\partial \phi_{2}\left(x\right)}{\partial t}=\frac{\partial }{\partial x}\;\Lambda_{21}\frac{\partial }{\partial x}\left(\frac{\delta F}{\delta \phi_{1}}\right)+\frac{\partial }{\partial x}\Lambda_{22}\frac{\partial }{\partial x}\left(\frac{\delta F}{\delta \phi_{2}}\right)$$

with F the free energy, $\frac{\delta F}{\delta \phi_{i}}$ the exchange chemical potentials and Λ_*ij*_ the elements of the Onsager mobility matrix. The definition of the latter is given in Section S1.2. (Supporting Information). The model is incompressible, which means that we may treat the glycerol fraction $\phi_{3}$ as a dependent variable, given by $\phi_{3}$ = 1 − $\phi_{1}$ − $\phi_{2}$. Following earlier work,^[^ ^42^, ^43^, ^44^ ^]^ we write the free energy of the PEDOT:PSS:glycerol mixture as a summation of local and non‐local contributions:

(3) $$F\;\left[\phi_{1},\phi_{2}\right]=\int dx\left[f_{\mathrm{loc}}\left(\phi_{1},\phi_{2}\right)+f_{\mathrm{nonloc}}\left(\partial_{x}\phi_{1},\partial_{x}\phi_{2}\right)\right]$$

where we define the local contribution based on Flory‐Huggins theory^[^ ^45^ ^]^:

(4) $$\begin{array}{ccc} \beta f_{\mathrm{loc}}\;\left(\phi_{1},\phi_{2}\right) & = & \frac{\phi_{1}}{N_{1}}\;\ln\phi_{1}+\frac{\phi_{2}}{N_{2}}\ln\phi_{2}+\phi_{3}\ln\phi_{3}+\chi_{12}\phi_{1}\phi_{2}\\ & & +\chi_{13}\phi_{1}\phi_{3}+\chi_{23}\phi_{2}\phi_{3} \end{array}$$

and $f_{\mathrm{nonloc}}\left(\partial_{x}\phi_{1},\partial_{x}\phi_{2}\right)$ the free energy penalty associated with the formation with concentration gradients:

(5) $$\begin{array}{ccc} \beta f_{\mathrm{nonloc}}\;\left(\partial_{x}\phi_{1},\partial_{x}\phi_{2}\right) & = & \frac{1}{2}\;\kappa_{11}{\left(\partial_{x}\phi_{1}\right)}^{2}+\kappa_{12}\partial_{x}\phi_{1}\partial_{x}\phi_{2}\\ & & +\frac{1}{2}\kappa_{22}{\left(\partial_{x}\phi_{2}\right)}^{2} \end{array}$$

with *β* = 1/*k_B_T* . In Equation (4) *N_i_* is an effective molecular size, normalized by that of the solvent, and $\chi_{ij}$ represent mean‐field binary interaction parameters. In Equation (5), $\kappa_{ij}$ are gradient energy coefficients,^[^ ^46^ ^]^ assumed concentration‐invariant for simplicity.

Based on Equation (4), we calculate an approximate ternary phase diagram (Figure 1b) for the PEDOT:PPS:glycerol‐mixture, using the following parametrization: *N*_1_ = 40, *N*_2_ = 200, $\chi_{12}$ = 0.065, and $\chi_{13}$ = $\chi_{23}$ = 0. These parameters are consistent with the notion that i) the PEDOT chains are shorter than the PSS chains, ii) the effective repulsion between PEDOT and PSS is high enough to make sure the components are (mostly) phase separated in the absence of glycerol and iii) the glycerol indeed acts as a compatibilizer by interacting favorably with both PEDOT and PSS. For simplicity, we assume the net interaction of the glycerol between both the PEDOT and the PSS to be athermal by taking $\chi_{13}$ = $\chi_{23}$ = 0. This choice is consistent with the fact that PEDOT:PSS is capable of accommodating substantial amount of glycerol.^[^ ^38^, ^39^ ^]^ A possible difference in the interaction (so $\chi_{13}$ ≠ $\chi_{23}$) would, given the fact that the total solids content is high, only result in a marginal tilt in the tie‐lines in Figure 1b. In other words, it would leave the results of the calculations, as well as the conclusions unaffected. We obtain the binodal curve (dark green) of coexisting compositions in the usual way by applying a Maxwell construction based on equalizing the chemical potentials and the osmotic pressures in the coexisting phases,^[^ ^46^ ^]^ which themselves are connected by the tie‐lines (grey). The spinodal curve (green), i.e., the limit of thermodynamic stability, is obtained through the condition: det(H) = 0, with H the Hessian matrix of second derivatives of the free energy with respect to the independent volume fractions. The (approximate) position of the critical point (light green) is obtained by interpolation.

The blue (PSS‐rich) and red (PEDOT‐rich) dots in Figure 1b indicate the out‐of‐equilibrium situation right after the initial glycerol ingress, but before the diffusive redistribution of the polymeric components (i.e., PEDOT and PSS), that takes place to set a new equilibrium. From top to bottom, the dots indicate exemplary average glycerol volume fractions of ${\bar{\phi }}_{3}$ = 0.55, 0.45, 0.35, and 0.25 in the PEDOT:PSS, for simplicity deemed the same as the fractions in the PVA, as mentioned above. As the system slowly relaxes toward the new equilibrium (represented by the binodal), the arrows show how the phase compositions change during the redistribution. Due to the asymmetry in the phase diagram, the PSS‐rich phase will during this process accommodate a significant amount of PEDOT, in particular at high glycerol content. In contrast, almost no PSS enters the PEDOT‐rich phase, represented by the fact that the red arrows are very short. Within our model, it is exactly the enrichment of PEDOT in the PSS‐rich phase that leads to enhanced connectivity between the PEDOT‐rich domains that we deem responsible for the increase in conductivity.

To demonstrate the actual dynamics of the change in composition in adjacent PEDOT‐ and PSS‐rich domains, we numerically integrate Equations (1) and (2) on a 250 nm wide box, comprising a PSS‐rich domain at the left side interfacing a PEDOT‐rich domain on the right (see Figure 1d), with initial compositions represented by the blue and red symbols in Figure 1b. We detail the exact calculation procedure in Section S1.3 (Supporting Information). Figure 1d shows the result for ${\bar{\phi }}_{3}$ = 0.55, plotting the normalized PEDOT concentration as a function of spatial coordinate and time. The results for the other values for ${\bar{\phi }}_{3}$ are given in Figure S3 (Supporting Information). As mentioned, more PEDOT migrates into the PSS‐rich phase than the other way around. Concomitantly, mass conservation causes the position of the interface between the domains, roughly indicated by the inflection point in the spatial profile of the PEDOT volume fraction across the domain, to shift from left to right. We schematically indicate this in Figure 1e.

To make a comparison with the measured sheet resistance transients in the upper panel of Figure 1c, we calculate the total amount of PEDOT in the PSS‐rich phase at each time step by integrating the PEDOT concentration profile between x = 0 and the position of the inflection point. The normalized value Φ (see Section S1.4., Supporting Information) is then plotted as a function of time in the lower panel of Figure 1c. Comparing the two panels shows that the decrease in the sheet resistance scales with time in nearly exactly the same way as the increase in the PEDOT content in the PSS‐rich phase and confirms a linear correlation after saturation (after 24 h) by plotting (1‐ Φ) against the normalized sheet resistance (see Figure S4, Supporting Information). This strongly supports our mechanistic hypothesis behind the conductivity increase. Moreover, the fact that we reproduce the curvature in the transient stage across the range in glycerol content with only one single value for the fundamental (monomeric) diffusivity (see Section S1.2., Supporting Information), means that the theory we invoke to describe the inter‐diffusion between the three blend components correctly captures the composition‐dependence of the plasticization of the PEDOT:PSS matrix.

In summary, we demonstrate that a plasticizer that ingresses a PEDOT:PSS film from an underlying substrate enhances the conductivity of the former in a thermodynamically controlled way. Using a multicomponent diffusion model based on Flory‐Huggins free energy we show that this increase in conductivity is established by a slow rearrangement and interdiffusion of both the PEDOT and the PSS after fast initial ingress of the plasticizer. This reorganization leads to an enhanced connectedness of the PEDOT‐rich domains, where the time dependence of the fraction of PEDOT in the PSS‐rich phase is similar to that of the sheet resistance.

**Figure 2:**
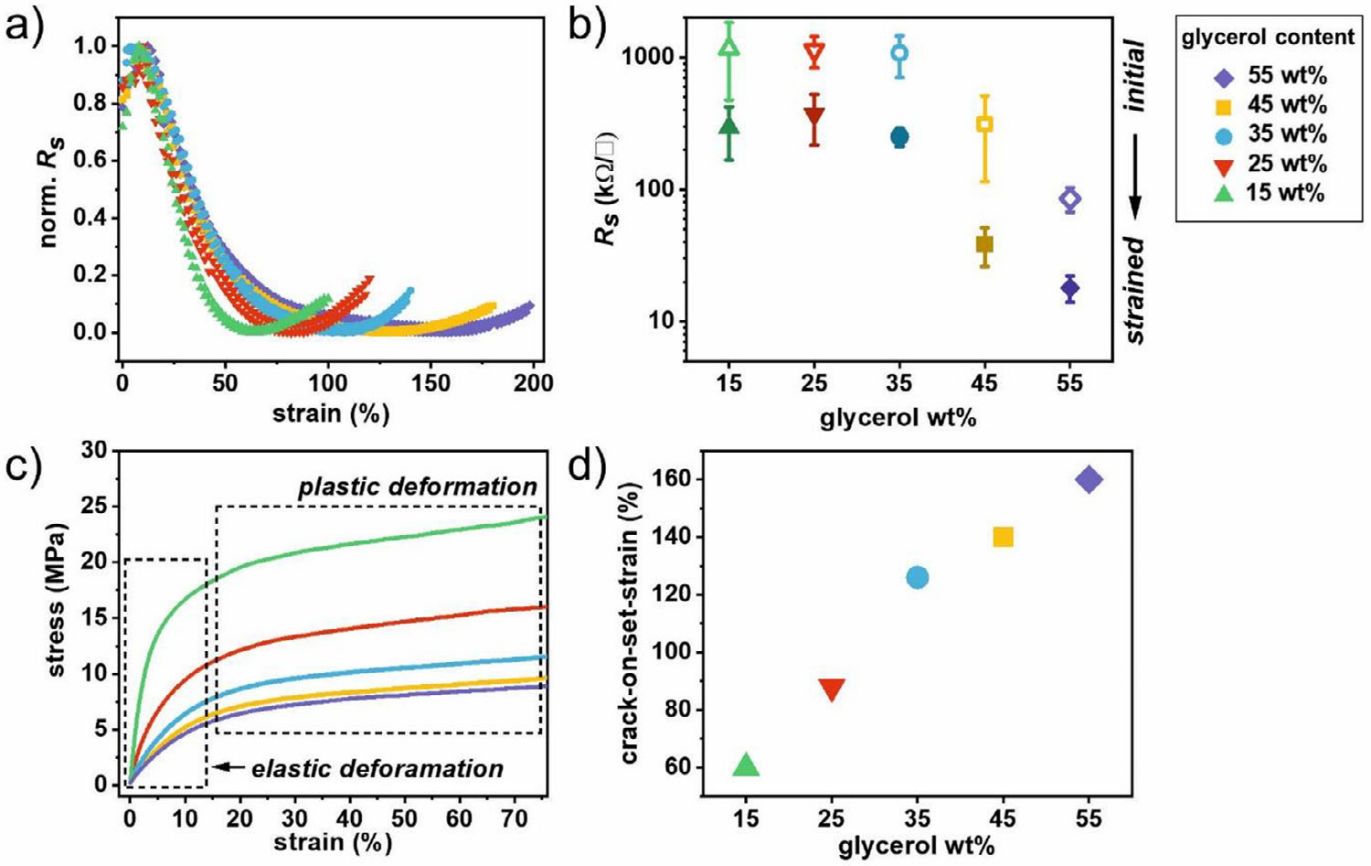
Electrical performance under strain: a) Normalized sheet resistance R_s_ of PEDOT:PSS films plotted against strain on PVA substrates containing 15–55 wt.% glycerol. b) Initial R_s_ (open symbols) compared to the minimum R_s_ in the strained state (filled symbols; 15 wt.% glycerol: 65% strain; 25 wt.%: 84%; 35 wt.%: 97%; 45 wt.%: 130%; 55 wt.%: 140%). c) Tensile tests conducted on pristine PVA:glycerol substrates. d) Crack‐on‐set‐strain for 15–55 wt.% glycerol.

After allowing PEDOT:PSS to reorganize in response to the glycerol diffusion, the change in electrical performance upon strain is studied. Accordingly, the films are clamped into an in house‐built stretch station and elongated in 2%‐steps at a speed of 0.06 mm s^−1^ to reach 100–200% strain in total. In contrast to other studies,^[^ ^19^, ^47^, ^48^, ^49^, ^50^ ^]^ we do not apply any kind of pre‐stretching to either the substrates or the PEDOT:PSS films. **Figure** **2**a depicts the change in min.‐max. normalized sheet resistance *R_s_* parallel to the direction of strain. Please see the Experimental Section and the Supporting Information for a detailed description of the sample analysis as well as the absolute *R_s_* and change in absolute resistance *R/R_0_* plotted against the strain (Figures S6 and S7, Supporting Information). Consistent with previous reports, we observe a decrease in sheet resistance upon strain. It is noteworthy that a decrease in resistance when stretching pristine spin‐coated PEDOT:PSS films has been reported to depend on the substrate.^[^ ^31^, ^32^, ^33^ ^]^ Using polyimide (PI) substrates, Lee et al.^[^ ^32^, ^33^ ^]^ attributed this observation to an irreversible growth of PEDOT grains and using SEBS substrates, Vijay et al.^[^ ^31^ ^]^ linked the slight decrease in resistance for up to 15% strain to a promoted adhesion to the substrate via van der Waals forces.

Looking at the normalized *R_s_* in dependence of the strain (Figure 2a), the following observations can be summarized: i) independently of the amount of glycerol incorporated into the substrates (15–55 wt.%), all curves share the same principle shape, which can be divided into three regimes. In the first regime they exhibit an increase in sheet resistance up to 10–20% strain. In the second regime they reach a minimum sheet resistance at 65–144% strain, depending on the glycerol amount (see also **Table** **1**). And in the third regime after the minimum the sheet resistance is increasing with further elongation. ii) The observed minima shift to higher strains with increasing glycerol amounts. iii) The overall decrease in sheet resistance upon stretching becomes more pronounced with an increasing glycerol amount (Figure 2b). The glycerol‐loading dependent effects indicate that the degree of glycerol penetration and plasticization varies within PEDOT:PSS. These observations are in line with the above findings. By incorporating a plasticizer into the underlying substrate, the mechanical properties of the substrate can be adjusted and, as a result of the mobility of the plasticizer between the systems, the ability of the conductive thin film to adapt to strain is improved. This approach provides dual benefits by improving both the flexibility of the substrate and the electrical performance of the thin film under strain simultaneously. In stark contrast to this approach, literature reports of impurities that potentially migrate from the substrate into the conductive layer, decreasing its electrical performance.^[^ ^6^ ^]^ Figure 2b depicts the averaged initial and minimum sheet resistances (*R_S_*) extracted from the data displayed in Figure S8 (Supporting Information). Not only does the initial *R_s_* decrease (consistent with the results presented and discussed in Figures 1 and 2a), but additionally the minimum *R_S_* shifts to lower values meaning that a higher glycerol loading leads to a higher conductivity at higher strains. The most substantial changes are observed for films containing 55 wt.% glycerol: Upon stretching *R_s_* reduces to one‐fifth of its initial value at 142% strain. In addition, films containing 55 wt.% glycerol show stable electrical properties when cyclically stretched within their elastic range (Figure S9, Supporting Information).

**Table 1:** Strain at which the minimum sheet resistance R_s_ and crack‐onset strain for PEDOT:PSS on PVA:glycerol (15–55 wt.%) substrates (average over ≥ 5 films.) are observed.

Glycerol amount	Min. *R_s_* at strain	Crack‐on‐set strain
15 wt.%	65%	60%
25 wt.%	84%	88%
35 wt.%	97%	126%
45 wt.%	130%	140%
55 wt.%	141%	160%

To better understand how changes in the sheet resistance of PEDOT:PSS relate to the rearrangement of the substrate during elongation, we conduct tensile tests on the pristine PVA:glycerol substrates (Figure 2c; Figure S10, Supporting Information). These tests reveal typical elastomeric behavior, starting with an elastic regime, transitioning to a plastic regime, followed by strain hardening and ultimately leading to sample failure. Interestingly, we observe that the initial increase in *R_s_* upon stretching (up to ≈20%, Figure 2a) aligns with the elastic deformation regime of the substrates. Within this regime, the PVA polymer chains remain unchanged in their arrangement. However, at higher strains in the plastic regime, the chains are expected to rearrange, forming new hydrogen bonds. The arrangement of the substrate during the plastic deformation regime may contribute to aligning the PEDOT:SS chains, potentially leading to a decrease in *R_s_* both parallel and perpendicular to the direction of strain.

**Figure 3:**
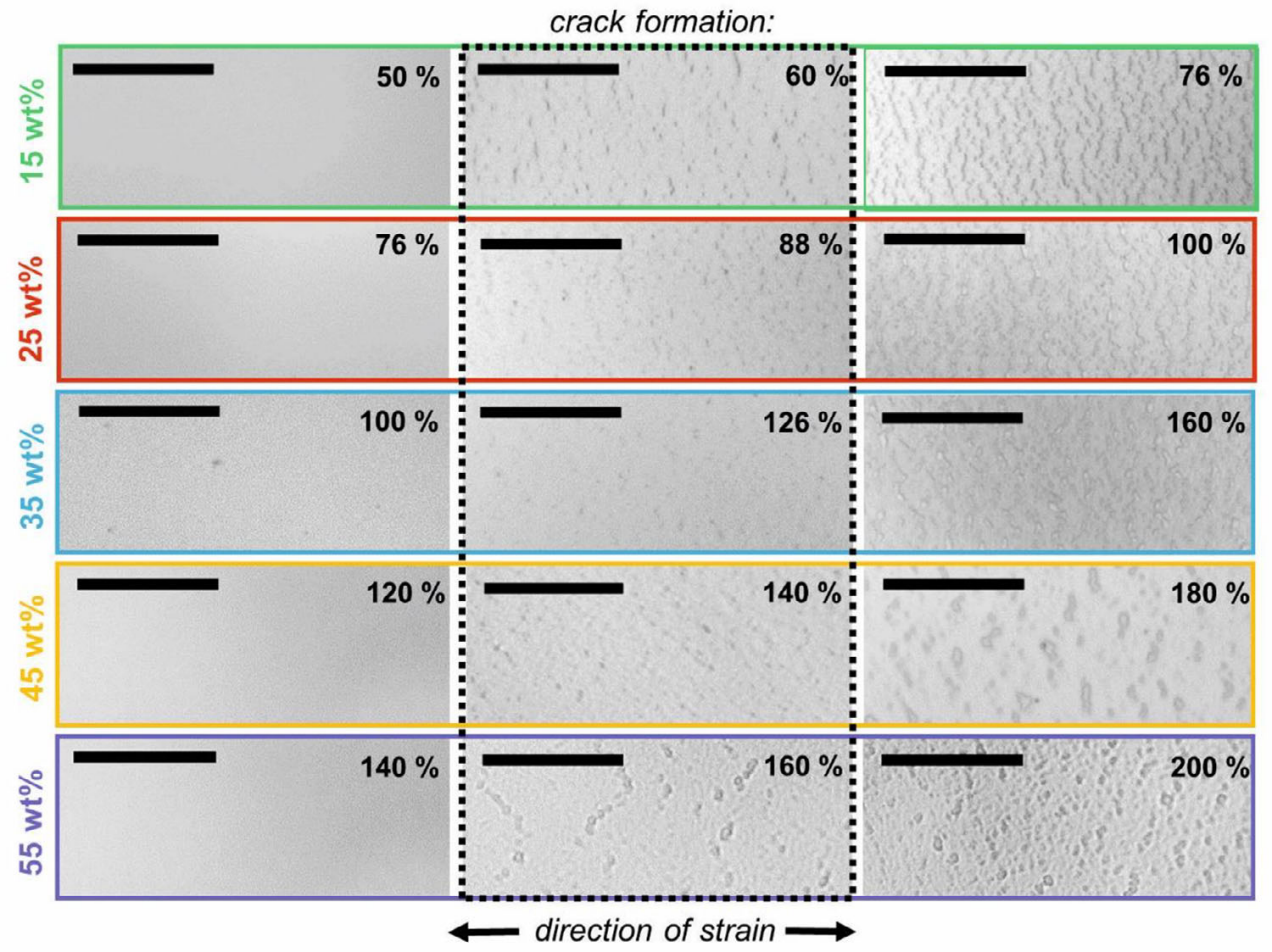
Crack formation of PEDOT:PSS on PVA:glycerol (15–55 wt.%) substrates at different strain percentages. All scale bars are 25 µm.

When stretching conductive materials, cracks often lead to a decrease in electrical performance, limiting their maximum elongation.^[^ ^6^, ^8^, ^19^, ^20^, ^21^, ^33^, ^34^ ^]^ Furthermore, crack formation may be dependent on the underlying substrate.^[^ ^8^, ^33^ ^]^ To address this issue, plasticizing surfactants and ionic liquids are frequently added to PEDOT:PSS.^[^ ^19^, ^20^, ^21^ ^]^ To study the crack formation (crack‐on‐set‐strain) we use optical microscope imaging and examine the films at different strains on a micrometer scale (see Table 1 and Figures 2d and **3**). Due to the glycerol's plasticizing effect within the PEDOT:PSS, cracks only emerge at higher strains with increasing glycerol content. In all substrates, the emergence of cracks is linked to the observed minimum in sheet resistance during stretching. This is expected, as crack formation leads to an increase in the sheet resistance. Slight variations in the onset strain of cracks and the percentage of strain at which a minimum in sheet resistance is observed may be attributed to slight irregularities in the films. Cracks, typically 2–5 µm in size and distributed evenly, increase in size and quantity after their initial appearance with increasing strain. An optical comparison of the cracks reveals an edge softening with increasing glycerol amount. The retention of conductivity despite cracking can be explained by the presence of sufficient intact conductive pathways, a phenomenon previously reported in the literature.^[^ ^6^, ^17^ ^]^ Finally, we would like to point out that using our glycerol‐loaded substrates no additional surfactant is needed to realize crack‐free elongation of PEDOT:PSS of up to 160%.

**Figure 4:**
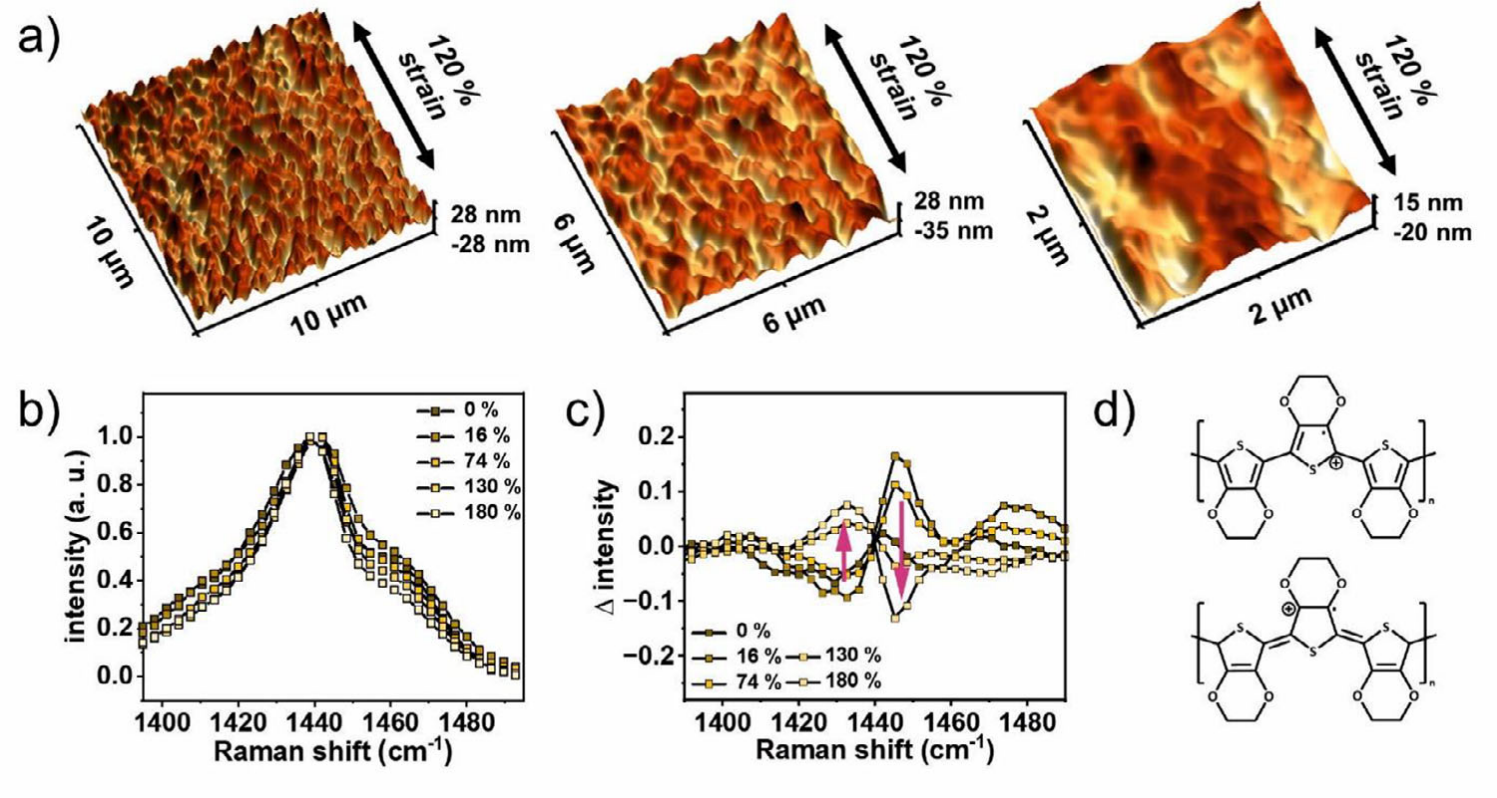
Properties of strained PEDOT:PSS thin films: a) SFM topography images of PEDOT:PSS on top of PVA:glycerol (55 wt.%) substrates, strained to 120%. b) BCARS spectra of PEDOT:PSS on PVA:glycerol (45 wt.%) at various strains. c) Subtracting BCARS spectra of blank PVA:glycerol (45 wt.%) from PEDOT:PSS on top of PVA:glycerol (45 wt.%) at different strains. d) Chemical structures of benzoid and quinoid resonant structures of PEDOT.

Scanning force microscopy (SFM) is employed in PeakForce Quantitative Nanomechanical mapping mode to investigate microscale changes. **Figure** **4**a depicts 3D topography images within three differently‐sized areas of PEDOT:PSS films on PVA:glycerol (55 wt.%) and elongated to 120% strain. Please note that the films are kept strained while being examined. 2D topography images can be found in Figure S11 (Supporting Information). The images reveal a valley‐like structure aligned with the direction of strain. In line with the microscope images in Figure 3 (bottom row), no cracks are observed. Upon comparing these images with topography recordings of the pristine PVA:glycerol substrates (55 wt.%) under strain, minimal differences are observed (see Figure S12, Supporting Information for 2D as well as 3D recordings). This suggests that the underlying substrate is primarily responsible for the observed topography.

To investigate changes on a molecular level, we record Broadband Coherent Anti‐Stokes Raman Scattering (BCARS) spectra of PEDOT:PSS on PVA: glycerol (45 wt.%) substrates at various strains. Please see the Supporting Information for a detailed description of the data analysis as well as all related figures (Figures S14 and S15 and Table S2, Supporting Information). In brief, we first analyze blank PVA:glycerol (45 wt.%) films, followed by those with transfer‐printed PEDOT:PSS. The spectra were then deconvoluted based on the characteristic peaks of the incorporated components (PEDOT:PSS, PVA, and glycerol). PEDOT:PSS was identified through the vibrational modes of its aromatic thiophene rings, corresponding to the quinoid (1438 cm⁻¹) and benzoid (≈1443 cm⁻¹) structures. PVA and glycerol were evidenced by their respective peaks at 1443 and 1463 cm⁻¹ (see also Table S2, Supporting Information).^[^ ^51^, ^52^, ^53^, ^54^, ^55^, ^56^, ^57^, ^58^, ^59^, ^60^, ^61^, ^62^ ^]^ And lastly, to isolate PEDOT‐specific changes, spectra were subtracted to eliminate overlapping contributions from PVA and glycerol. For the PEDOT:PSS‐transfer‐printed film (Figure 4b), at lower strains (16%), we observed an initial blue shift of the main peak (≈1440 cm⁻¹) and an increase in the right shoulder (≈1460 cm⁻¹), which aligns with changes detected in pristine PVA:glycerol films (Figure S14a, Supporting Information). However, at higher strains (≥74%), this trend is reversed, with a redshift of the main peak from 1443 to 1438 cm⁻¹ and a reduction in the right shoulder (1443 and 1463 cm⁻¹ sub‐peaks). Subtracting the spectra, as described above, revealed a shift in PEDOT's resonant structure: a decrease in the benzoid fraction (1443 cm⁻¹) and an increase in the quinoid fraction (1438 cm⁻¹; red arrows in Figure 4c).^[^ ^53^ ^]^ This change is typically associated with enhanced charge transport due to the extended‐coil arrangement of the quinoid structure^[^ ^14^, ^23^, ^54^ ^]^ (see Figure 4d for chemical structures). While this change is not immediately visible in Figure 4b due to overlapping contributions from PVA and glycerol, it becomes evident upon in Figure 4c. The change in resonant structure most likely is supported by the PVA chain‐alignment, which is identified when examining blank PVA films (Figure S14a–c, Supporting Information). Additionally, it is noteworthy that the above‐described effects become more pronounced with higher glycerol content, e.g. when incorporating 45 wt.% glycerol compared to 15 wt.% (comparing Figures S14 and S15, Supporting Information). In conclusion, these measurements reveal molecular‐level changes in PEDOT:PSS under strain, driven by diffusion and glycerol‐induced plasticization.

## 3 Conclusion

In conclusion, we introduce a novel, straight‐forward and broadly applicable approach for stretchable electronic materials and devices by utilizing the ability of a plasticizer incorporated into the polymer substrate to diffuse into a conductive polymer film (PEDOT:PSS). We closely investigate the interaction between the conductive polymer thin film (PEDOT:PSS), a plasticizer (glycerol) and a supporting substrate (PVA) in a stretchable bilayer system. Detailed insights into how a diffusion‐active organic plasticizer induces structural reorganization within PEDOT:PSS, which subsequently leads to increased conductivity and improved strain adaptability, is gained using a multi‐component diffusion model incorporating a Flory‐Huggins free energy of mixing. A loading‐dependent enhanced intermixing effect is found to be responsible for the observed effects. The effect of plasticization is then investigated in detail using various techniques such as electrical characterization, microscopic imaging, SFM, and Raman spectroscopy. The improved adaptability to strain leads to a decrease in resistance upon stretching, which is accompanied by a higher onset strain for crack formation and strain‐induced PEDOT chain‐alignment. With increasing glycerol loading the effects become more pronounced. Incorporating 55 wt.% glycerol into the underlying substrate we are able to remarkably decrease the sheet resistance to 20% of its initial value while simultaneously shifting the crack‐onset‐strain to 140%. This fundamental study provides insights into plasticization of PEDOT:PSS and its resulting effects on electrical and morphological properties. Furthermore, it establishes novel approaches toward ductile conducting materials that are applicable in stretchable devices such as OECTs.

## 4 Experimental Section

### Materials

PEDOT:PSS (Clevios PH1000, Heraeus Deutschland GmbH), glycerol (PanReac AppliChem, 99% for synthesis), *iso*‐propanol and acetone (Honeywell Riedel‐de haën), PVA (M_w_ = 31 000–50 000 g mol^−1^, 99% hydrolyzed; Sigma Aldrich) were used without further purification.

### Sample Preparation

All samples were prepared and examined under controlled humidity (*R_H_* = 40–45%) and temperature (*T* = 22 °C) conditions. Sample preparation involved cleaning glass slides (2.5 × 7.5 cm) in an ultrasonic bath with deionized water (5 min), acetone (10 min) and iso‐propanol (10 min), followed by drying at 140 °C for 10 min. Subsequently, the slides were treated in an UV/ozone oven for 20 min. Prior to spin‐coating, the PEDOT:PSS suspension was kept in an ultrasonic bath for 20 min and filtered (45 µm filter). Spin‐coating was performed at 750 rpm for 60 s, and the resulting thin films were dried at 120 °C for 10 min. PVA:glycerol substrates were prepared by combining the components in DI water, stirring at 90 °C for 3 h, and subsequent cooling to room temperature. The mixture was then filtered, treated in an ultrasonic bath (10 min), and 1.5 mL of the solution was drop casted onto pre‐cleaned glass slides, allowing it to dry. The PEDOT:PSS thin films then were transfer‐printed heatless as described in detail in Ref. [36] the average thickness of the PEDOT:PSS thin films was determined to be 70–75 nm using a surface profilometer (Bruker, DektakXT Stylus Profiler) and an average over six films.

### Diffusion Coefficients

Due to the complexity of the data analysis, the reader is referred to a detailed description in Section S1 (Supporting Information).

### Electrical Characterization

The conductivity (σ) was calculated using the equation σ = *l*/(*R* · *w* · *t*). with the length *l*, the resistance *R*, the width *w* and the thickness *t*. The sheet resistance was calculated using the following equation: *R_s_* = 1/(σ · *t*) = *R* · *w*/*l*. Before stretching, the films were repeatedly contacted to measure the resistance over a period of up to 48 h. Electrical measurements upon stretching were performed using a home‐built setup based on a motorized stretch station synchronized with a Keithley 2000 Multimeter. Gold‐coated clamps were utilized to enable good electrical and mechanical contact during stretching. The films were elongated in ramps with 2% strain at each step with respect to the initial length and in both directions uniformly. The stretching time was set to 10 s, resulting in a speed of 0.06 mm s^−1^. The wait time at each ramp was 30 s (see Figure S5, Supporting Information for graphical visualization). Depending on the supporting substrate, 50–100 ramps were performed, resulting in an overall strain of 100–200% with the initial length being set to 0%. One second after the beginning and 1 s before the end of each performed stretching and waiting period a resistance value was recorded. Data was normalized using a min‐max normalization.

### Imaging—Optical Microscopy

Microscopic images were recorded in bright field mode and edited using a confocal microscope (Leica DM6000 M) and the according software (Leica Application Suite Version 4.12.0). The strained films were secured on glass slides using double‐sided tape.

### Imaging—SFM

PeakForce‐Quantitative Nanomechanical Mapping (PF‐QNM) SFM measurements were performed using a Bruker Dimension ICON platform using OLTESPA NanoAndMore cantilevers (frequency: 70 KHz, spring constant: 2 N/m, back/ tip side coating: Al). Images recorded in strained state were subject to Fourier transformation to eliminate the periodicity induced by the film's vibration during the measurement.

### Imaging—BCARS Spectroscopy

Due to the complexity of data recording and evaluation, the reader is referred to a detailed description in Section S4 (Supporting Information).

### Tensile Tests

Tensile tests were performed on a materials testing machine Z005 (Zwick/Roell, Germany). Bone shape films with 4.5 mm width, 32 mm length, and thicknesses of ≈0.1 mm were elongated with a rate of 20 mm min^−1^ at room temperature. A pre‐load of 0.1 N was used in all experiments and stress versus strain characteristics were recorded.

## Conflict of Interest

The authors declare no conflict of interest.

## Author Contributions

U.K. proposed the project. C.V. and U.K. developed the concept and designed the experiments. J.J.M. conceptualized the model and performed the calculations. C.V. carried out the experiments. R.C. helped with the electrical characterization. M.B. and P.G.A. performed the Raman spectroscopy and analysis. U.K. supervised the project. C.V., J.J.M. and U.K. wrote the original draft of the manuscript with input from all authors. All authors discussed the results and reviewed and edited the manuscript.

## Supporting information



Supporting Information

## Data Availability

The data that support the findings of this study are available from the corresponding author upon reasonable request.
