# Supplementary material for: Enhanced Electrical Performance and Stretchability by Plasticizer‐Facilitated PEDOT:PSS Self‐Alignment
Source: Adv Sci (Weinh). 2025 May 8;12(27):2502853. doi: 10.1002/advs.202502853 (PMC12279167; doi:10.1002/advs.202502853)
Supplement: Supplementary file 1 — Supporting Information [file ADVS-12-2502853-s001.pdf]

ADVANCED SCIENCE
Open Access

Supporting Information

for *Adv. Sci.*, DOI 10.1002/advs.202502853

Enhanced Electrical Performance and Stretchability by Plasticizer-Facilitated PEDOT:PSS Self-Alignment

Carla Volkert, Mateusz Brzezinski, Pablo Gomez Argudo, Renan Colucci, Sapun H. Parekh, Pol Besenius, Jasper J. Michels and Ulrike Kraft**

Supplementary information:

Enhanced Electrical Performance and Stretchability by Plasticizer-facilitated PEDOT:PSS Self-alignment

Carla Volkert[1], Mateusz Brzezinski[2,3], Pablo Gomez Argudo[3], Renan Colucci[1], Sapun H. Parekh[3,4], Pol Besenius[5], Jasper J. Michels[2*], Ulrike Kraft[1*]

[1]Organic Bioelectronics Research Group, Max Planck Institute for Polymer Research, 55128 Mainz, Germany
[2]Department of Molecular Electronics, Max Planck Institute for Polymer Research, 55128 Mainz, Germany
[3]Department of Molecular Spectroscopy, Max Planck Institute for Polymer Research, 55128 Mainz, Germany
[4] Department of Biomedical Engineering, University of Texas Austin, Austin, TX, USA 78712
[5]Department of Chemistry, Johannes Gutenberg University Mainz, 55128 Mainz, Germany

Contents

1. Diffusion of glycerol
    1.1 Experimental curve fitting
    1.2 Onsager mobility matrix
    1.3 Calculation procedure polymer concentration profiles
    1.4 Improving accuracy and determining PEDOT content in the PSS-rich phase
2. Electrical characterization
3. Tensile tests
4. Scanning Force Microscopy
5. Raman spectroscopy
6. Additional references

1. Diffusion of glycerol

The set of data showed in Fig. S1 was collected and analyzed to understand the glycerol-triggered PEDOT and PSS re-arrangement. To achieve this, the sheet resistance of all eight films within each dataset was averaged and then min-max normalized ($x' = (x - x_{min})/(x_{max} - x_{min})$) relative to the averaged minimum of the film containing 55 wt% glycerol. The obtained data is displayed on the bottom right (Fig. S1f; identical to Fig. 1c top main paper).

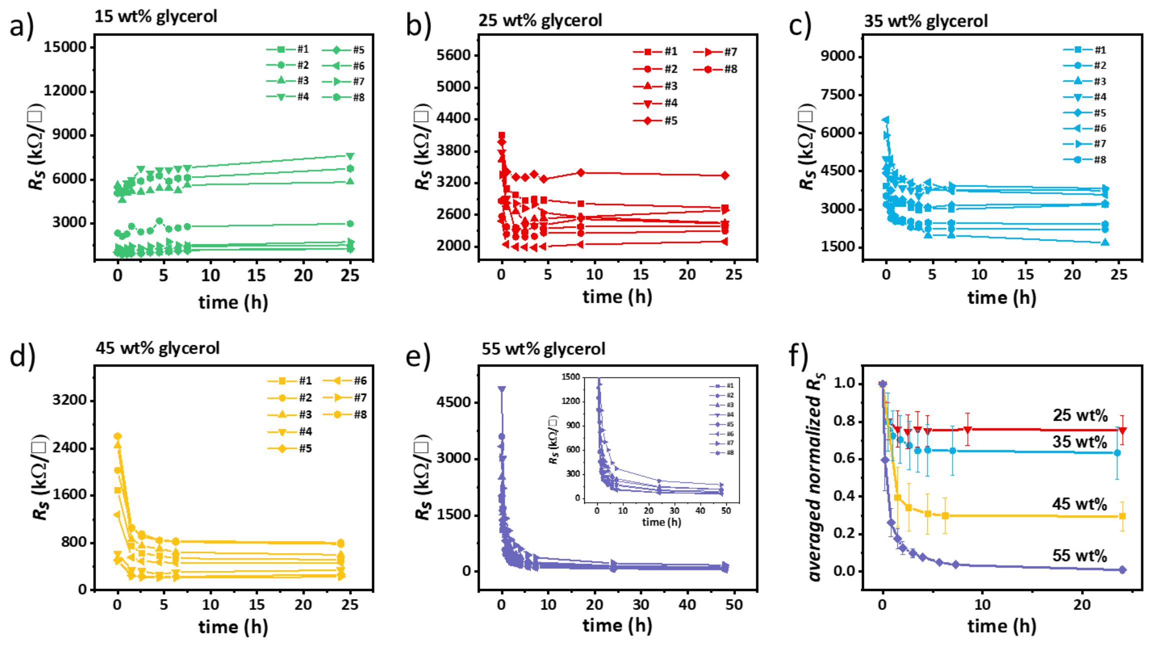

***Figure S1**: a-e) Time-dependent change in sheet resistance R_S of PEDOT:PSS on PVA substrates containing 15, 25, 35, 45 and 55 wt% glycerol (0 % strain). f) Averaged normalized sheet resistances over time of transfer-printed PEDOT:PSS on glycerol-loaded PVA substrates.*

1.1 Experimental curve fitting

To test whether the transient increase in conductivity of the PEDOT:PSS could be directly related to actual diffusion of glycerol from the PVA substrate into the PEDOT:PSS film, we fitted the normalized resistivity versus time data to the following empirical exponential function to extract a characteristic resistivity decay time τ:

$$f(t) = (1 - y_0)\exp\left(-\frac{t}{\tau}\right) + y_0 \tag{S1}$$

, with t indicating time and y_0 a constant off-set. The experimental data and fits are shown in Fig. S1. The obtained values for y_0 and τ are listed in Table S1. As mentioned in the main text, despite the fact that the fits have reasonable quality, the obtained values for τ is for all glycerol loading orders of magnitude higher than what would be expected if the decrease in resistivity were dominated by the actual diffusive ingress of glycerol from the PVA into the PEDOT:PSS. Illustratively, if we, for simplicity, assume an effective film thickness of the order $\bar{l}$ = 10^2 nm, we find effective diffusivities $D_{EFF} = \bar{l}^2/\tau$ in the range: 10^{-18} – 10^{-19} m^2/s. Indeed, this fast ingress causes an initial swelling of the PEDOT:PSS film, which occurs too fast to allow for measurement.

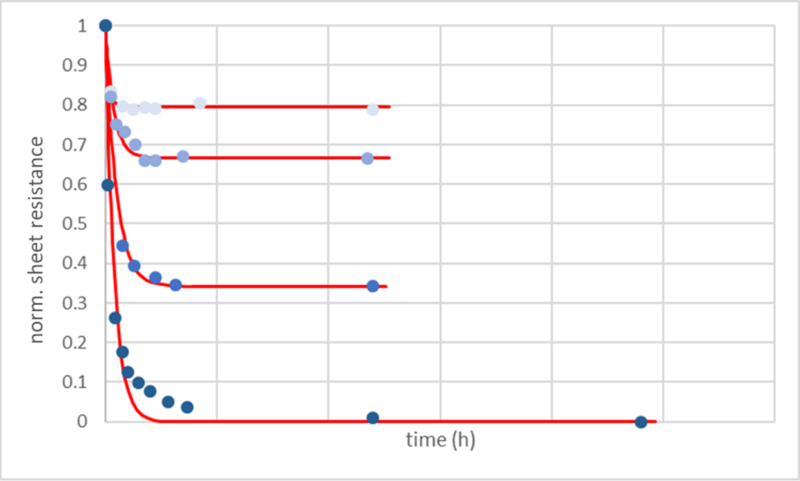

Figure S2. *Normalized sheet resistance plotted as a function of time. Points are measured data and lines are fits based on Equation S1. The shades of blue correspond to substrate glycerol loadings of 25%, 35%, 45% and 55% from light to dark.*

Table S1: *Fit values obtained from fitting Equation S1 to the experimental resistivity versus time data.*

glycerol loading	τ (hours)	y_0
25%	0.3	0.795
35%	0.75	0.666
45%	1.0	0.342
55%	0.8	0

1.2 Onsager mobility matrix

As in previous work[1–3], the Cahn-Hilliard simulations in this work utilize the fast mode diffusion formalism[4] to correctly implement mass transport under the constraint of incompressibility. For an n-component mixture the mobility coefficients are given by:

$$\Lambda_{ij}(\mathbf{r},t) = \phi_i(\mathbf{r},t)\phi_j(\mathbf{r},t)\sum_{k=1}^{n}\lambda_k + \left(\delta_{ij} - \phi_i(\mathbf{r},t)\right)\lambda_j - \phi_j(\mathbf{r},t)\lambda_i \quad \text{(S2)}$$

, with $\lambda(\mathbf{r},t)$ the diagonal elements of the Onsager mobility matrix. For component i we write:

$$\lambda_i(\mathbf{r},t) = \phi_i(\mathbf{r},t)N_i D_i \quad \text{(S3)}$$

, with N_i and D_i molecular size and tracer diffusivity. Assuming free draining, Rouse-like mass transport, the latter is related to that of a fundamental monomeric diffusivity D_0 according to: $D_i = D_0/N_i$. As before, we neglect the off-diagonal contributions of the Onsager mobility matrix[5]. With a fixed value of D_0 = 2×10^{-4} µm^2/s, we reproduce the experimentally observed kinetics for the increase in conductivity with time.

1.3 Calculation procedure polymer concentration profiles

We evolved the concentration profiles of PEDOT ($\phi_1(x,t)$) and PSS ($\phi_2(x,t)$) in time by numerically solving the Flory-Huggins-Cahn-Hilliard Equations (1) and (2) (main text) in one space dimension on a domain $\tilde{X}$ containing 50 grid points $0 \le \tilde{x} \le 49$, with a grid spacing of 5 nm (so in dimensional units: $0 \le x \le 250$ nm). The initial concentration profiles of PEDOT (component 1), PSS (component 2) and glycerol (component 3) are predefined by the following expressions:

$$\phi_{i=1,2}(\tilde{x},0) = \frac{1}{2}\left[\phi_i^{(\infty)}(0) - \phi_i^{(-\infty)}(0)\right]\left\{1 + \tanh\left[a\left(\tilde{x} - \frac{1}{2}\tilde{X}\right)\right]\right\} + \phi_i^{(-\infty)}(0) \quad \text{(S4)}$$

$$\phi_3(\tilde{x},0) = 1 - \phi_1(\tilde{x},0) - \phi_2(\tilde{x},0) = \text{const} \quad \text{(S5)}$$

, with $\phi_i^{(\infty)}(0)$ and $\phi_i^{(-\infty)}(0)$ the initial volume fractions in the two phases, *i.e.* right after swelling. These are given by:

$$\phi_{i=1,2}^{(-\infty)}(0) = \left(1 - \phi_3^{(-\infty)}(0)\right)\phi_i^{(\alpha)} \quad \text{(S6)}$$

$$\phi_{i=1,2}^{(\infty)}(0) = \left(1 - \phi_3^{(\infty)}(0)\right)\phi_i^{(\beta)} \quad \text{(S7)}$$

, with $\phi_3^{(\mp\infty)}(0)$ the initial (given) volume fractions of glycerol in the PEDOT- and PSS rich phase, taken as $\phi_3^{(-\infty)}(0) = \phi_3^{(\infty)}(0)\epsilon\{0.2,0.3,0.4,0.5\}$, as explained in the main text. $\phi_i^{(\alpha)}$ and $\phi_i^{(\beta)}$ represent the binodal volume fractions of PEDOT and PSS before swelling with glycerol in, respectively, the PSS- and PEDOT rich phases. The composition of the 'dry' phases is given by the ternary phase diagram for ϕ_3 = 0. We have: $\phi_1^{(\alpha)}$ = 0.054; $\phi_2^{(\alpha)}$ = 0.946; $\phi_1^{(\beta)}$ = 0.996; $\phi_2^{(\beta)}$ = 0.004. The stiffness coefficients are taken sufficiently high to ensure that the interface between the coexisting phases is described by a minimum of ~5 grid points. We assume that only the two polymers contribute measurably to the stiffness in the concentration fields due to the typically large entropic penalty near the interface, which does not occur for small molecular species. For simplicity we assume the same stiffness parameter for both polymers: $\frac{\kappa_{11}}{k_B T} = \frac{\kappa_{22}}{k_B T}$ = 1.0 nm^2 and $\frac{\kappa_{12}}{k_B T}$ = 0.

Starting with the initial conditions given above, the materials are allowed to redistribute upon relaxation to equilibrium. The numerical simulations are performed on a time domain of 128000 s, using a time step of 0.05 s. To solve Equations (1) and (2) we used a forward-time-central-space (FTCS) explicit finite difference scheme. Every 64 s, the concentration fields are stored. To determine the total amount of PEDOT in the PSS-rich phase as a function of time, we integrate each stored PEDOT concentration profile $\phi_1(\tilde{x},t)$ in the interval $0 \le \tilde{x} \le \tilde{x}_s(t)$, with $\tilde{x}_s(t)$ the position of the inflection point. The latter is determined by the maximum in the numerical derivative of composition with respect to coordinate.

1.4 Improving accuracy and determining PEDOT content in the PSS-rich phase

In order to improve the accuracy in the determination of the position of the inflection point, as well as the spatial integration of the concentration profile (see below) without having to increase the number of calculated points against considerable computational cost, we interpolate additional points between each pair of adjacent calculated compositions using the Matlab cubic spline interpolation routine spline().m. Figure S3 shows for $\phi_3^{(-\infty)}(0) = \phi_3^{(\infty)}(0) = 0.25, 0.35, 0.45, 0.55$ the bare calculated concentration profiles (points), as well as the interpolated curves as a function of time (lines). To obtain the amount of PEDOT in the PSS-rich phase, we numerically integrate the interpolated PEDOT concentration profiles between position x = 0 and x = $x_I(t)$, with $x_I(t)$ the position of the inflection point as a measure for the position of the interface between the PSS- and PEDOT-rich phase. This position coincides with the maximum in the first spatial derivative (orange curves). The latter is determined using the Matlab function diff().m. To facilitate the comparison with the experimentally measured conductivity curves, we normalized the integrated time profiles for the different glycerol levels by the value obtained for the dry film (*i.e.* 0% glycerol). The data was then normalized by the plateau (equilibrium) value of the film with the highest (55%) glycerol content.

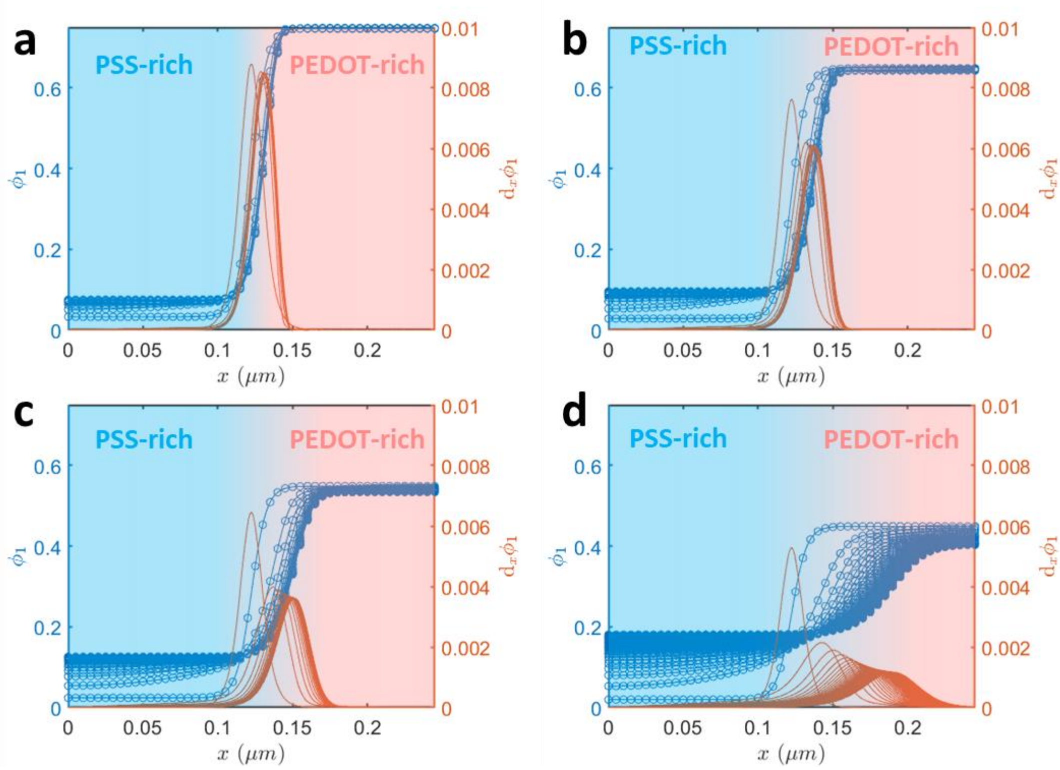

Figure S3. *Calculated and interpolated PEDOT volume fractions, plotted as a function of time and lateral coordinate, assuming* $\phi_3^{(-\infty)}(0) = \phi_3^{(\infty)}(0) = 0.25, 0.35, 0.45, 0.55$ *(panel a – d). The open circles represent the calculated data, obtained by numerically solving Cahn-Hilliard Equations (1) and (2) (see main text) on a 50-point grid. The blue lines are obtained by a cubic spline interpolation. The inflection point in the concentration profiles is given by the maximum of the first spatial derivative of the interpolated curves (orange curves).*

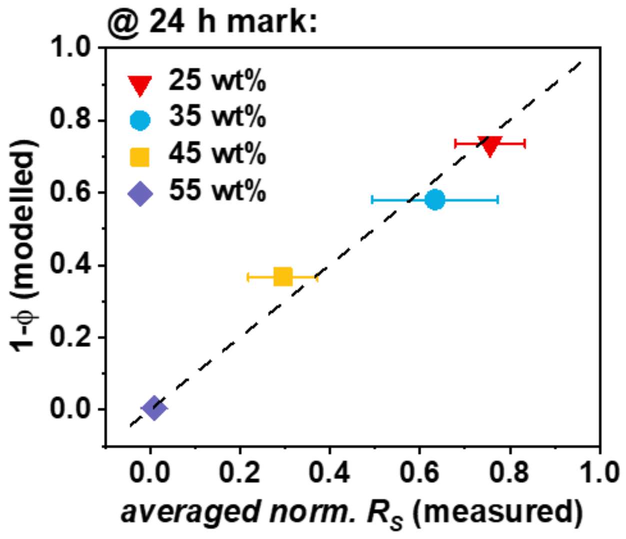

Fig. S4: *Normalized fraction of PEDOT in the PSS rich phase (derived from modelling) vs. averaged normalized sheet resistances (measured) for PEDOT:PSS on PVA-substrates containing 25 – 55 wt% glycerol. Both sets of values were extracted from the data presented in Fig. 1c at the 24-hour time mark. For improved clarity, the data derived from modelling (ϕ) is plotted as (1- ϕ).*

2. Electrical characterization

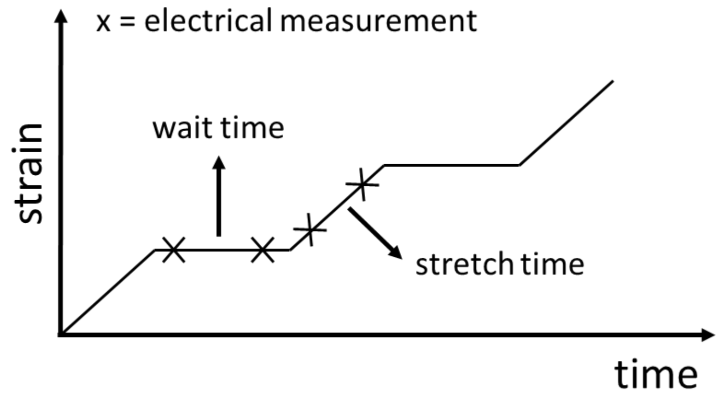

Figure S5*: Graphical illustration of elongation process. 'x' indicates the recording of electrical data. This means that for each straining step two data points were recorded. Furthermore, we only strained in the forward direction.*

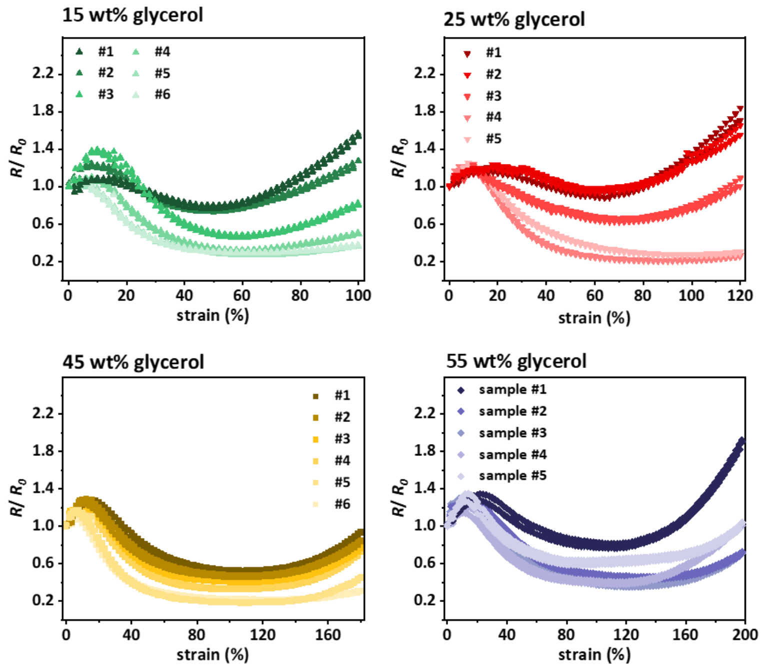

Figure S6: *Change in resistance (R/R_0) upon stretching of PEDOT:PSS on PVA substrates containing 15, 25, 35, 45 or 55 wt% glycerol.*

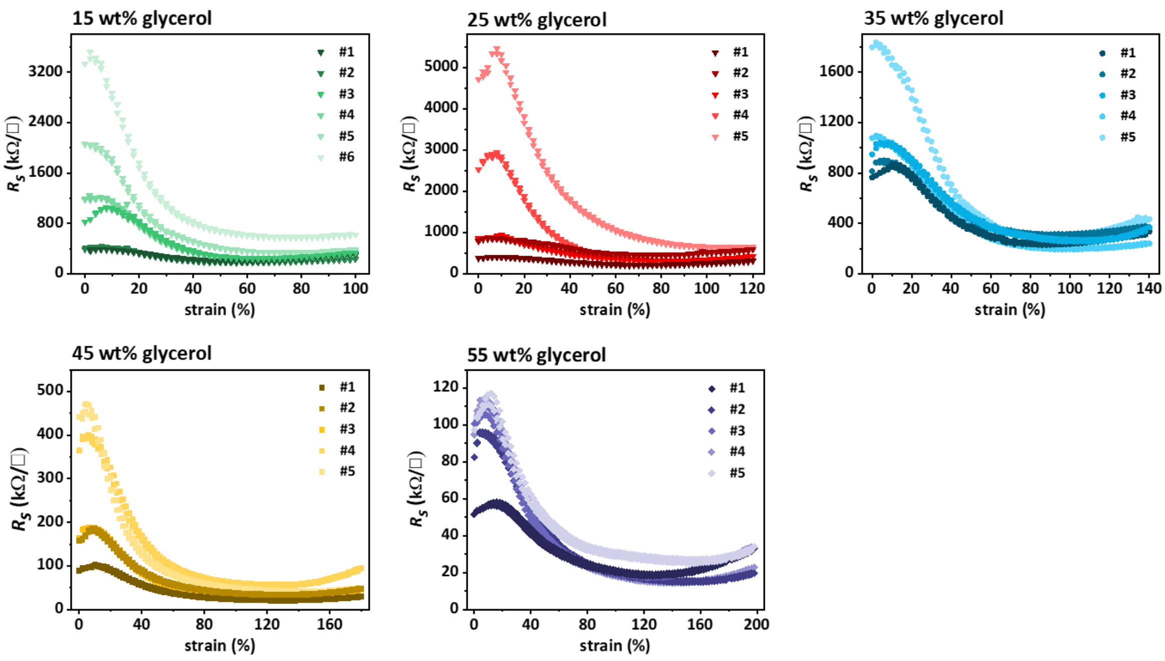

Figure S7: *Sheet resistance (R_s) upon stretching of PEDOT:PSS on PVA substrates containing 15, 25, 35, 45 or 55 wt% glycerol.*

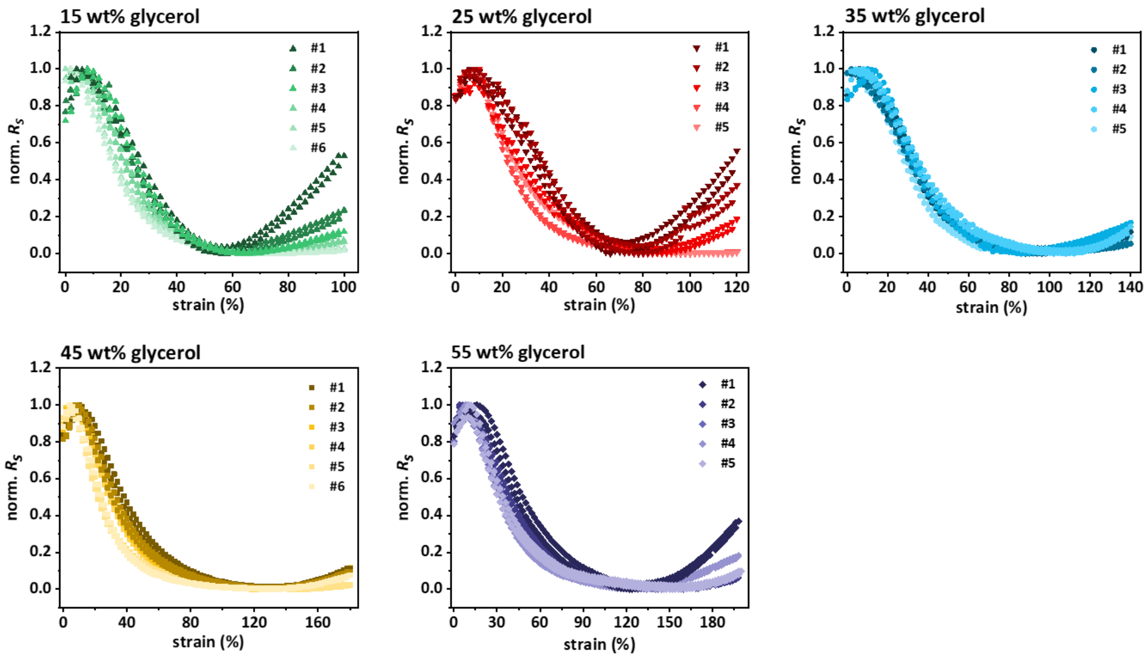

Figure S8: *Min.-max. normalized sheet resistance (R_s) upon stretching of PEDOT:PSS films on PVA substrates containing 15, 25, 35, 45 or 55 wt% glycerol.*

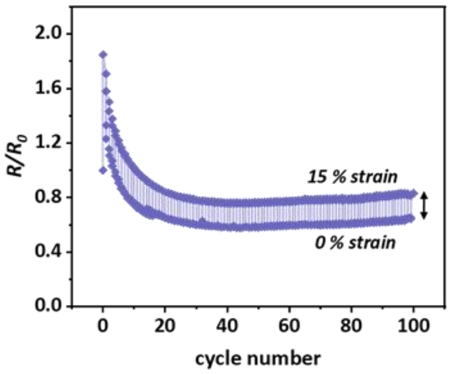

Figure S9: *R/R_0 for repeated stretching cycles in the elastic range of PVA:glycerol (55 wt%) substrates. The films were stretched 100 times to 15% elongation (speed: 0.15 mm/s; wait time in elongated and returned state: 30 sec).*

3. Tensile tests

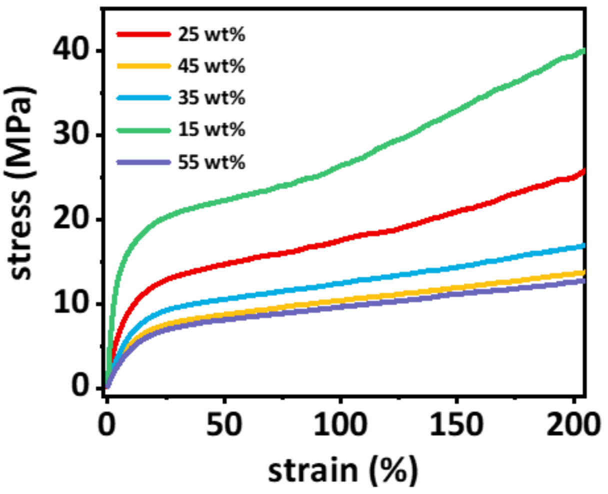

Figure S10: *Stress-strain curves for PVA:glycerol substrates at different weight percentages of glycerol (15, 25, 35, 45, and 55 wt%).*

4. Scanning Force Microscopy

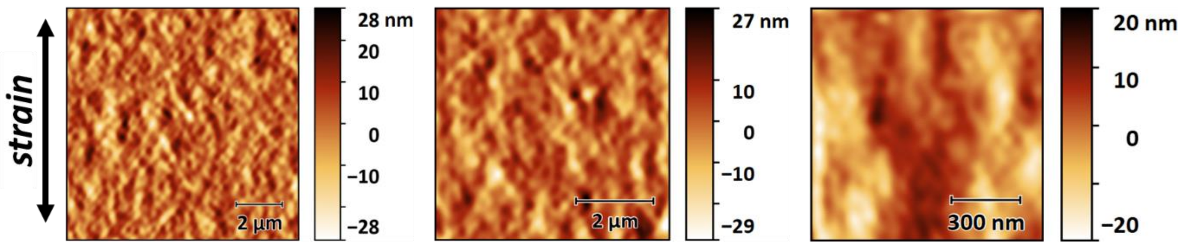

Figure S11: *2D topography images of PEDOT:PSS films on PVA:glycerol (55 wt%) substrates at 120 % strain. Areas from left to right: 10 x 10 µm, 6 x 6 µm and 2 x 2 µm.*

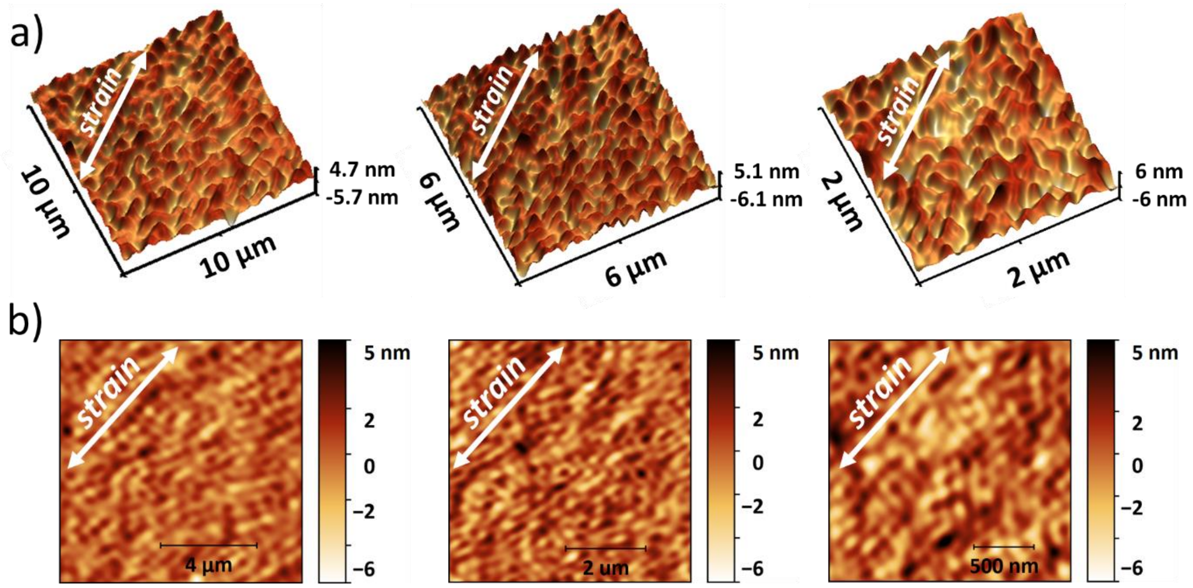

***Figure S12**: a) 2D and b) 3D topography images of PVA:glycerol (55 wt%) substrates at a strain of 120 %. Areas from left to right: 10 x 10 µm, 6 x 6 µm and 2 x 2 µm.*

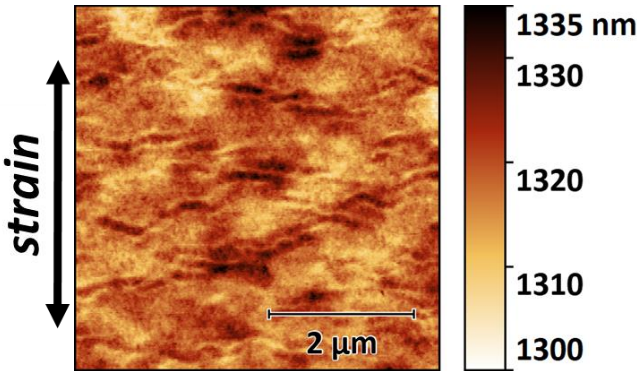

***Figure S13**: 2D topography images of PEDOT:PSS films transfer-printed onto PVA:glycerol (45 wt%), strained to 108 % and examined after release.*

5. Raman spectroscopy

Data acquisition and analysis: Raman measurements were performed using a home-built broadband coherent anti-Stokes Raman scattering (BCARS) microscope, described in detail elsewhere.[6] Briefly, the pump/probe and Stokes pulses are generated in a dual-output subnanosecond laser source (CARS-SM-30, Leukos). These pulses are then synchronously superimposed in space and time at the sample plane of an inverted microscope (Eclipse Ti-U, Nikon) and tightly focused onto the sample using a 0.85 NA air objective (LCPlan N, Olympus). The BCARS signal, once isolated from the excitation pulses, is focused onto the slit of a spectrograph (Shamrock 303i, Andor). And the dispersed spectral components finally reach a cooled CCD camera (Newport DU920P-BR-DD, Andor). Samples were positioned with the coverslip facing the collector and scanned using a piezo stage (Nano-PDQ 375 HS, Mad City Labs) controlled by LabView 2015 software (National Instruments). The collected hyperspectral data were processed in IgorPro (WaveMetrics). The Raman-like spectra were retrieved

through a modified Kramers-Kronig transform.[7] All spectra presented here were phase-retrieved using glass alone, without macromolecules as a reference spectrum, and any background phase is removed using a Savitzky-Golay filter with a 2nd order polynomial and a window size of approximately 400 cm^{-1}. This protocol allowed the acquisition and interpretation of high-quality spectral data. For the final spectra analysis and deconvolution in the 1390 to 1490 cm^{-1} range, a custom Python script was developed. This script was instrumental in generating initial parameter seeds for the Lorentzian sub-peaks, derived from the normalized spectra within the specified range. To obtain these initial parameters, characteristic peaks specific to PEDOT:PSS, PVA and glycerol were manually identified, fed into the peak analysis module of the OriginLab Pro software and further refined for the deconvolution process, thereby achieving the final resolution of each peak. Details of the deconvolution process, including the parameters used, are given in Table S1.

Experimental setup: Blank PVA:glycerol samples containing either 15 wt% or 45 wt% glycerol and samples with attached PEDOT:PSS were elongated and affixed to glass slides using double-sided tape. Considering that the PEDOT:PSS film is substantially thinner (approximately 1:1000) than the supporting PVA:glycerol substrate and both layers exhibit signals in the Raman broadband range (1380 - 1480 cm^{-1}), a comprehensive sample analysis was conducted. For each sample, spectra were obtained, deconvoluted and classified into five sub-peaks, representing PVA, glycerol and PEDOT:PSS if present. The assignments are as follows: artificial sub-peak positions collectively describing aromatic thiophene rings of PEDOT: 1412 cm^{-1} and 1434 cm^{-1}, PEDOT:PSS-quinoid structure: 1438 cm^{-1}, PEDOT:PSS-benzoid structure as well as PVA (vibration mode): 1443 cm^{-1} and glycerol (vibration mode): 1463 cm^{-1} [8,9,18,19,10–17] (see also Table S2). Following deconvolution, the peaks were integrated, subtracted from one another and plotted against the strain. Please note that spectra recorded at 0 % strain were omitted from analysis due to substantial material transformations at minimal strains, not allowing for a reliable data analysis. This finding might provide an additional explanation for the initially observed dip in conductivity for up to 20 % strain in Fig 2a. Nevertheless, we believe that the exclusion of unstrained samples from our analysis does not compromise the validity or the integrity of the observed trends. Fig. S10 summarizes the obtained spectra for samples with 45 wt% glycerol, while spectra for samples with 15 wt% are provided in Fig. S11.

Analyzing blank strained PVA:glycerol (both 15 wt% and 45 wt%) substrates resulted in a blue shift of the maximum and left concave-upwards Raman band. This led to a decrease in the area of sub-peaks at 1412 cm^{-1} and 1434 cm^{-1}, coupled with an increase in the area of sub-peaks at 1438 cm^{-1} and 1443 cm^{-1} (Fig S10 a-c and S11 a-c).

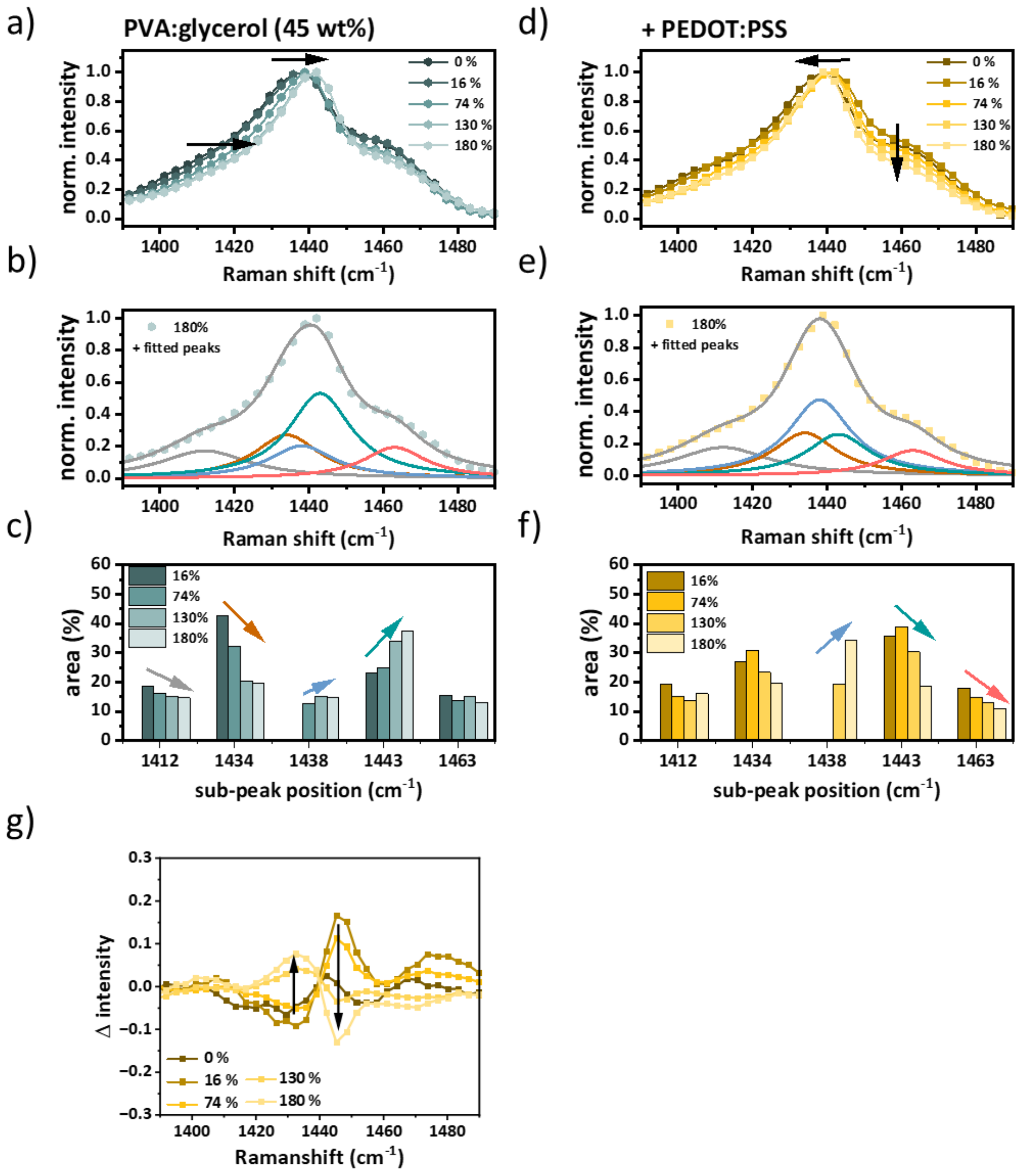

***Figure S14**: BCARS studies of PEDOT:PSS on PVA:glycerol (45 wt%) substrates. **Left**: Examining the blank PVA:glycerol (45 wt%) substrates: a) BCARS spectra of blank PVA:glycerol (45 wt%) at various strains. b) fitted and deconvoluted spectra at 180 % strain. c) Area changes in deconvoluted sub-peak position for each strain. **Right**: Examining PEDOT:PSS atop PVA:glycerol (45 wt%) substrates: d) BCARS spectra of PEDOT:PSS atop PVA:glycerol (45 wt%) at various strains. e) fitted and deconvoluted spectra at 180 % strain. f) Area changes in deconvoluted sub-peak position for each strain. **Bottom**: g) Subtracting BCARS spectra of blank PVA:glycerol (45 wt%) from PEDOT:PSS on top of PVA:glycerol (45 wt%) at different strains.*

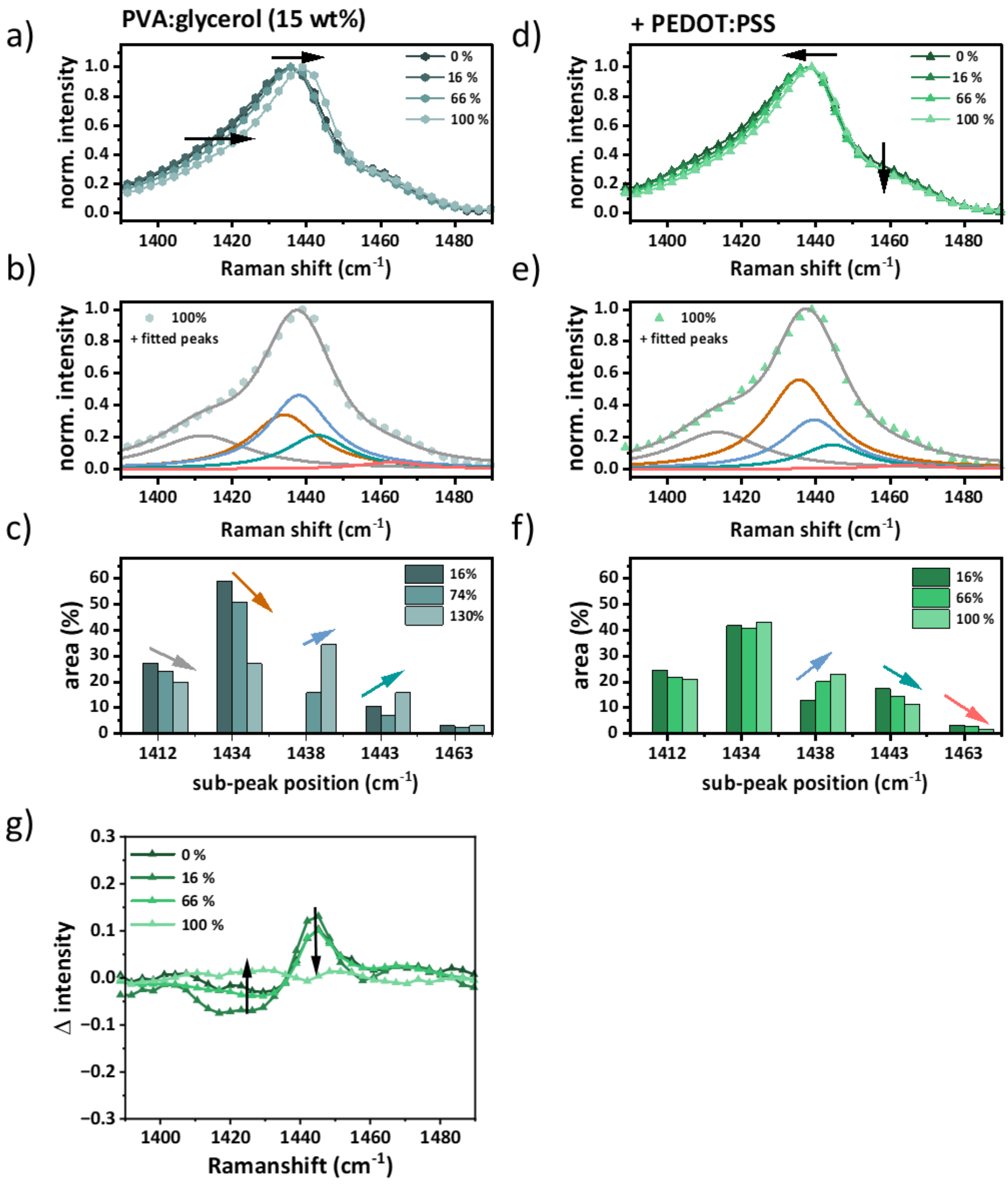

***Figure S15**: BCARS studies of PEDOT:PSS on PVA:glycerol (15 wt%) substrates. **Left**: Examining the <u>blank PVA:glycerol (15 wt%) substrates</u>: a) BCARS spectra of blank PVA:glycerol (15 wt%) at various strains. b) fitted and deconvoluted spectra at 100 % strain. c) Area changes in deconvoluted sub-peak position for each strain. **Right**: <u>Examining PEDOT:PSS atop PVA:glycerol (15 wt%) substrates</u>: d) BCARS spectra of PEDOT:PSS atop PVA:glycerol (15 wt%) at various strains. e) fitted and deconvoluted spectra at 100 % strain. f) Area changes in deconvoluted sub-peak position for each strain. **Bottom**: g) Subtracting BCARS spectra of blank PVA:glycerol (15 wt%) from PEDOT:PSS on top of PVA:glycerol (15 wt%) at different strains.*

***Table S2**: Seed parameters for BCARS peak deconvolution with initial position, initial FWHM and initial area plus freedom each.*

sub-peak number	1	2	3	4	5
**initial position**	1412 cm^{-1}	1434 cm^{-1}	1438 cm^{-1}	1443 cm^{-1}	1463 cm^{-1}
**position freedom**	± 0 cm^{-1}	± 0 cm^{-1}	± 0 cm^{-1}	± 0 cm^{-1}	± 0 cm^{-1}
**initial FWHM**	35 cm^{-1}	25 cm^{-1}	25 cm^{-1}	25 cm^{-1}	25 cm^{-1}
**freedom of initial FWHM**	± 0 cm^{-1}	± 0 cm^{-1}	± 0 cm^{-1}	± 0 cm^{-1}	± 0 cm^{-1}
**initial area**	0 %	0 %	0 %	0 %	0 %
**area freedom**	∞ %	∞ %	∞ %	∞ %	∞ %

6. Additional references

[1] M. M. Abolhasani, M. Naebe, K. Shirvanimoghaddam, H. Fashandi, H. Khayyam, M. Joordens, A. Pipertzis, S. Anwar, R. Berger, G. Floudas, J. Michels, K. Asadi, *Nano Energy* **2019**, *62*, 594.

[2] H. Soleymani, M. Noormohammadi, M. A. Kashi, M. H. Amiri, J. J. Michels, K. Asadi, M. M. Abolhasani, *Adv. Mater. Interfaces* **2021**, *8*, 2001734.

[3] H. S. Dehsari, J. J. Michels, K. Asadi, *J. Mater. Chem. C* **2017**, *5*, 10490.

[4] E. J. Kramer, P. Green, C. J. Palmstrøm, *Polymer (Guildf).* **1984**, *25*, 473.

[5] C. Schaefer, J. J. Michels, P. Van Der Schoot, *Macromolecules* **2016**, *49*, 6858.

[6] N. Billecke, G. Rago, M. Bosma, G. Eijkel, A. Gemmink, P. Leproux, G. Huss, P. Schrauwen, M. K. C. Hesselink, M. Bonn, S. H. Parekh, *Histochem. Cell Biol.* **2014**, *141*, 263.

[7] C. H. Camp Jr., J. S. Bender, Y. J. Lee, *Opt. Express* **2020**, *28*, 20422.

[8] H. Yu, Z. Wu, X. Huang, S. Shi, Y. Li, *Org. Electron.* **2018**, *62*, 121.

[9] L. Van Gheluwe, E. Munnier, H. Kichou, K. Kemel, F. Mahut, M. Vayer, C. Sinturel, H. J. Byrne, F. Yvergnaux, I. Chourpa, F. Bonnier, *Molecules* **2021**, *26*.

[10] S. H. Chang, C. H. Chiang, F. S. Kao, C. L. Tien, C. G. Wu, *IEEE Photonics J.* **2014**, *6*, 1.

[11] F. C. Tang, J. Chang, F. C. Wu, H. L. Cheng, S. L. C. Hsu, J. S. Chen, W. Y. Chou, *J. Mater. Chem.* **2012**, *22*, 22409.

[12] C. Hou, H. Yu, *J. Mater. Chem. C* **2020**, *8*, 4169.

[13] M. Kong, M. Garriga, J. S. Reparaz, M. I. Alonso, *ACS Omega* **2022**, *7*, 39429.

[14] S. Sakamoto, M. Okumura, Z. Zhao, Y. Furukawa, *Chem. Phys. Lett.* **2005**, *412*, 395.

[15] M. Stavytska-Barba, A. M. Kelley, *J. Phys. Chem. C* **2010**, *114*, 6822.

[16] B. D. Saksena, *Proc. Indian Acad. Sci. (Math. Sci.)* **1939**, *10*, 333.

[17] N. A. Zubair, N. A. Rahman, H. N. Lim, R. M. Zawawi, Y. Sulaiman, *RSC Adv.* **2016**, *6*, 17720.

[18] Y. A. Badr, K. M. Abd El-Kader, R. M. Khafagy, *J. Appl. Polym. Sci.* **2004**, *92*, 1984.

[19] W. Zhen, C. Lu, C. Li, M. Liang, *Appl. Clay Sci.* **2012**, *57*, 64.
